# *Mangifera indica* Fruit Extract Improves Memory Impairment, Cholinergic Dysfunction, and Oxidative Stress Damage in Animal Model of Mild Cognitive Impairment

**DOI:** 10.1155/2014/132097

**Published:** 2014-01-29

**Authors:** Jintanaporn Wattanathorn, Supaporn Muchimapura, Wipawee Thukham-Mee, Kornkanok Ingkaninan, Sakchai Wittaya-Areekul

**Affiliations:** ^1^Department of Physiology, Faculty of Medicine, Khon Kaen University, Khon Kaen 40002, Thailand; ^2^Integrative Complementary Alternative Medicine Research and Development Center, Khon Kaen University, Khon Kaen 40002, Thailand; ^3^Faculty of Pharmaceutical Sciences, Naresuan University, Phitsanulok 65000, Thailand

## Abstract

To date, the effective preventive paradigm against mild cognitive impairment (MCI) is required. Therefore, we aimed to determine whether *Mangifera indica* fruit extract, a substance possessing antioxidant and cognitive enhancing effects, could improve memory impairment, cholinergic dysfunction, and oxidative stress damage in animal model of mild cognitive impairment. Male Wistar rats, weighing 180–200 g, were orally given the extract at doses of 12.5, 50, and 200 mg*·*kg^−1^ BW for 2 weeks before and 1 week after the bilateral injection of AF64A (icv). At the end of study, spatial memory, cholinergic neurons density, MDA level, and the activities of SOD, CAT, and GSH-Px enzymes in hippocampus were determined. The results showed that all doses of extract could improve memory together with the decreased MDA level and the increased SOD and GSH-Px enzymes activities. The increased cholinergic neurons density in CA1 and CA3 of hippocampus was also observed in rats treated with the extract at doses of 50 and 200 mg*·*kg^−1^ BW. Therefore, our results suggested that *M. indica*, the potential protective agent against MCI, increased cholinergic function and the decreased oxidative stress which in turn enhanced memory. However, further researches are essential to elucidate the possible active ingredients and detail mechanism.

## 1. Introduction

To date, mild cognitive impairment (MCI), an intermediate state between normal cognition and dementia, is considered as a major public health problem due to its high socioeconomic burdens. It has been estimated that the number of dementia cases is approximately 81.1 million within 2040 [1]. Since MCI is a prodromal phase of dementia, its prevalence is also expected to be increased. Despite its increasing importance, the therapeutic efficacy is still very limited. Moreover, accumulative lines of evidence over the past decade have pointed out that preventive strategy is the most effective strategy in delaying or avoiding further cognitive decline. Therefore, the preventive paradigm which is cheap and easy to approach has gained much attention.

Experimental and clinical studies have demonstrated the involvement of the cholinergic system in cognitive function of animals and humans. Cholinergic hypofunction either via the structural lesion or via the application of muscarinic and nicotinic receptor antagonists could induce memory impairment [2]. Moreover, recent positron emission tomography (PET) data also show that cholinergic dysfunction is an early hallmark even before the onset of dementia at the clinical stage of MCI [3]. Besides cholinergic hypofunction, oxidative stress elevation also plays a crucial role in the pathophysiology of MCI. It has been reported that the level of malondialdehyde (MDA) is increased in this condition [4, 5]. In addition, substances possessing antioxidant activity can improve cognitive function of elderly patients suffering from MCI [6]. On the basis of crucial roles of oxidative stress and cholinergic system on MCI mentioned above, the protective effect against MCI with substances targeting at enhancing cholinergic function and decreasing oxidative stress has gained attention.

*Mangifera indica* or mango, a plant in a family of Anacardiaceae, has been for a long term used in traditional folklore. The medicinal properties of mango appear to be varied depending on the parts of mango tree and its cultivar. According to the traditional folklore, ripe mango fruit is believed to be invigorating and freshening. Its juice is reputed for restorative tonic and antiheat stroke [7]. In addition, mango is also regarded as a valuable dietary source of many phytochemical compounds which provide health benefit for the nervous system [8]. Recently, ethnaolic extract of mango fruit is reported to improve age-related memory deficit and memory deficit induced by scopolamine [9]. Based on the roles of cholinergic dysfunction and oxidative stress on the memory impairment and the beneficial effect of ethanolic extract of mango fruit on memory impairment, the protective effect of ethanolic extract of *Mangifera indica* (var. Nam Dok Mai) to improve memory deficit, cholinergic dysfunction, and oxidative stress in animal model of MCI has been focused on. In this study, we aimed to determine the effect of the ethanolic extract of ripe fruit of *M. indica* (var. Nam Dok Mai) on spatial memory of cognitive deficit rat induced by AF64A, a cholinotoxin. To further explore the possible underlying mechanism of extract, the effects of the extract both on the density of cholinergic neurons and on the oxidative stress markers including the level of malondialdehyde (MDA) and the activities of scavenger enzymes such as superoxide dismutase (SOD), catalase (CAT), and glutathione peroxidase (GSH-Px) in hippocampus, a brain area playing a crucial role on spatial memory, were also carried out.

## 2. Materials and Methods

### 2.1. Drugs and Chemicals

Donepezil hydrochloride (Aricept 5 mg/tablet) (Pfizer Pharmaceuticals Inc.) and vitamin C (500 mg/tablet) (Government Pharmaceutical Organization) were used as positive control in this study. They were dissolved in propylene glycol and administered via oral route. The animals were administered donepezil hydrochloride and vitamin C at doses of 1 and 250 mg/kg BW, respectively. All chemical substances used in this study were of analytical grade.

### 2.2. Animals

Adult male Wistar rats (180 ± 20 g, 8 weeks old) were obtained from National Laboratory Animal Center, Salaya, Nakhon Pathom, and they were housed in group of 5 per cage in standard metal cages at 22 ± 2°C on 12 : 12 h light-dark cycle. All animals were given access to food and water ad libitum. The experiments were performed to minimize animal suffering in accordance with the internationally accepted principles for laboratory use and care of European Community (EEC directive of 1986; 86/609/EEC).

The experimental protocols were approved by the Institutional Animal Care and Use Committee.

### 2.3. Plant Material and Preparation

Ripe fruits of *M. indica* (var. Nam Dok Mai) were purchased from local market in Amphoe Muang, Khon Kaen Province, Thailand, and were processed immediately after their arrival at the laboratory. All mangoes were free from physical and pathological cleaned and dried. The pulp of ripe mango fruit was homogenized and dried with freeze dryer for 3 days. Then, the dried homogenate was extracted with 95% ethanol at a ratio of 85 : 1 (gm : liter) by maceration technique. The yielded extract was filtered and the filtrate was dried at 40°C using rotary evaporator. The percent yield of the final extract was approximately 6.91. The concentrations of total phenolic compounds and beta-carotene in the fruit extract were 513.79 ± 27.17 mg of gallic acid equivalent (GAE)/mg fruit weight and 47.21 *μ*g/g, respectively.

### 2.4. Experimental Protocol

After acclimatization, rats were divided into 8 groups comprising 8 animals in each group as follows.

*Group I*. Control: rats in this group were exposed to sham operation plus vehicle (distilled water). 

*Group II*. Vehicle + ACSF: all animals in this group received vehicle or distilled water treatment at a period of 2 weeks before and 1 week after the intracerebroventricular (icv) administration of artificial cerebrospinal fluid (ACSF) bilaterally. 

*Group III*. Vehicle + AF64A: rats had been treated with vehicle for 2 weeks before and 1 week after the intracerebroventricular administration of AF64A, a cholinotoxin, in order to mimic a cholinergic deficit condition as observed in MCI. 

*Group IV*. Donepezil + AF64A: animals were orally treated with donepezil, a cholinesterase inhibitor which was used as standard drug for dementia treatment. The animals were treated in the same pattern as that mentioned in group III and they were served as positive control in this study. 

*Group V*. Vitamin C + AF64A: Rats were orally given vitamin C, a standard antioxidant which was previously reported to enhance memory and to attenuate neurodegeneration. This group was also treated in the same pattern as mentioned earlier in group III and they were served as positive control. 

*Groups VI–VIII*. MJ + AF64A: the animals were orally treated with the *M. indica* fruit extract at doses of 12.5, 50, and 200 mg·kg^−1^ BW, respectively (these doses were selected based on the doses of extract which showed the cognitive enhancing effect from our preliminary data).

The spatial memory 1 week after AF64A administration were determined; then they were sacrificed and determined the density of survival neurons in various subregions of hippocampus.

### 2.5. AF64A Administration

AF64A was prepared as described previously elsewhere [10]. Briefly, an aqueous solution of acetylethylcholine mustard HCl (Sigma, St. Louis, MO) was adjusted to pH 11.3 with NaOH. After the stirring for 30 min at room temperature, the pH of the solution was lowered to 7.4 with the gradual addition of HCl. Then, the solution was stirred for 60 min. The amount of AF64A was then adjusted to 2 nmol/2 *μ*L. The vehicle of AF64A was distilled water prepared in the same manner as the AF64A and recognized as ACSF. In order to administer AF64A bilaterally via intracerebroventricular (icv) route, the animals were anesthetized with the intraperitoneal injection of sodium pentobarbital at dose of 60 mg·kg^−1^ BW. Then, AF64A (2 nmol/2 *μ*L) was infused bilaterally via intracerebroventricular (icv) route with a 30-gauge needle inserted through a burr hole drilled into the skull into both the right and left lateral ventricles. Stereotaxic coordinates were (from the bregma) posterior 0.8 mm, lateral ±1.5 mm, and ventral (from dura) 3.6 mm. The rate of infusion was 1.0 *μ*L/min. The needle was left in place for 5 min after infusion and it was slowly withdrawn.

### 2.6. Morris Water Maze Test

The water maze consists of a metal pool (170 cm in diameter × 58 cm tall) filled with tap water (25°C, 40 cm deep). The pool was divided into 4 quadrants (NE, NW, SE, and SW) by two imaginary lines crossing the center of the pool. The removable platform was immersed below the water level and covered with a nontoxic milk powder at the center of one quadrant and remained there throughout training. The rats must memorize the platform location in relation to various environmental cues because there was nothing that directly showed the location of the platform in and outside the pool. Therefore, the placement of the water tank and platform was the same in all acquisition trials. Each rat was gently placed in the water facing the wall of the pool from one of the four starting points (N, E, S, or W) along the perimeter of the pool and the animal was allowed to swim until it found and climbed onto the platform. During training session, the rat was gently placed on the platform by experimenter when it could not reach the platform in 60 s. In either case, the subject was left on the platform for 15 s and removed from the pool. The time for animals to climb onto the hidden platform was recorded as escape latency. In order to determine the capability of the animals to retrieve and retain information, the platform was removed 24 hr later and the rat was released into the quadrant diagonally opposite to that which contained the platform. Time spent in the region that previously contained the platform was recorded as retention time. Prior to Morris water maze testing, all rats were habituated to swimming and they were trained with 4-trial shaping procedures with a 20 min intertrial interval for 3 days. In each trial, the animal was quickly dried with towel before being returned to the cage [11]. All tests were carried out within 45 minutes after the administration of vehicle or plant extract or vitamin C or donepezil, a cholinesterase inhibitor, which served as positive control.

### 2.7. Histological Procedure

Following anesthesia with sodium pentobarbital (60 mg·kg^−1^ BW), fixation of the brain was carried out by transcardial perfusion with fixative solution containing 4% paraformaldehyde in 0.1 M phosphate buffer pH 7.3. The brains were removed after perfusion and stored over a night in a fixative solution that was used for perfusion. Then, they were infiltrated with 30% sucrose solution for approximately 4°C. The specimens were frozen rapidly and 30 *μ*M thick sections were cut on cryostat. They were rinsed in the phosphate buffer and picked up on slides coated with 0.01% of aqueous solution of a high molecular weight poly L-lysine.

### 2.8. Choline Acetyl Transferase (ChAT) Immunohistochemistry

A series of sections containing hippocampus from each groups were reacted in parallel experiments using a mouse monoclonal antibody detected against choline acetyltransferase (ChAT) (Chemicon Internation, Inc., CA, USA) and a modification of a previously described protocol employing the DAKO Strept ABC Complex/HRP duet kit. In brief, the sections were eliminated endogenous peroxidase activity by 0.5% H_2_O_2_ in methanol. Sections were washed in running tap water and distilled water for 1 min each and then rinsed in KPBS and KPBS-BT for 5 min per each process. Then, the sections were incubated for 30 min in a blocking solution composed of 5% normal horse serum in KPBS-BT. After the blocking process, they were incubated in mouse primary antibody against ChAT diluted 1 : 100 in KPBS-BT at room temperature for 2 hours and then incubated at 4°C for 48 hours. The tissue was rinsed in KPBS-BT (two washes × 7 min), incubated for 4 hours in biotinylated goat antimouse IgG antibody, rinsed in KPBS-BT (two washes × 7 min), and then incubated in Strep ABC Complex/HRP for 4 hours. In the preparation for visualization step, sections were rinsed in KPBS-BT (1 min) and KPBS (two washes × 10 min). ChAT immunoreactivity was visualized using 0.025% 3, 3′ diaminobenzidine (DAB, Sigma) and 0.01% H_2_O_2_. Finally, sections were rinsed in running tap water, air-dried, and cover-slipped using permount.

### 2.9. Determination of Scavenger Enzymes Activities and Lipid Peroxidation Product

At the end of study period, all rats were sacrificed by cervical dislocation. Hippocampus was isolated and prepared as hippocampal homogenate and the determinations of malondialdehyde (MDA) or the lipid peroxidation product level and the activities of superoxide dismutase (SOD), catalase (CAT), and glutathione peroxidase (GH-Px) enzymes in hippocampus were performed. MDA level was estimated by determining the accumulation of thiobarbituric acid reactive substances (TBARS) in the brain homogenate. The activities of SOD, CAT, and GSH-Px were determined by recording the ability to inhibit cytochrome C, the rate of decrease in H_2_O_2_, and the amount of reduced nicotinamide adenine dinucleotide phosphate (NADPH) oxidized per minute, respectively [12].

### 2.10. Statistical Analysis

Data were presented as mean ± standard error of mean (SEM). One-way analysis of variance (ANOVA) was performed. In addition, the significant difference between the experimental groups and the other groups was followed by Tukey's *post hoc* test. Probability levels less than 0.05 were accepted as significance.

## 3. Results

### 3.1. Effect of *M. indica *Fruit Extract on Spatial Memory

**Figure 1:**
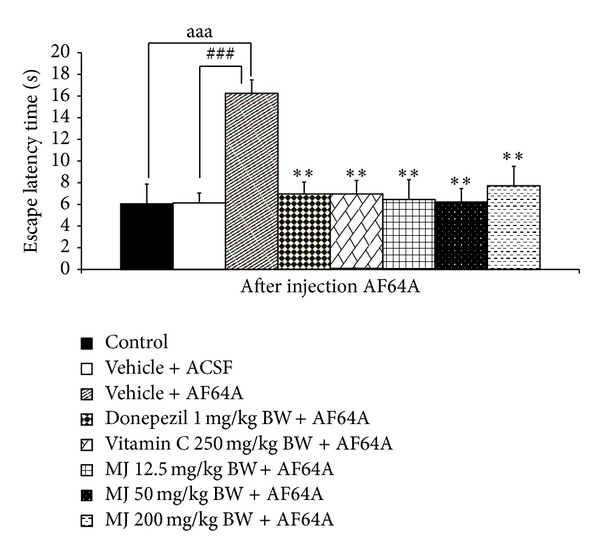
Effect of alcoholic extract of *M. indica* fruit on escape latency in Morris water maze test (*n* = 8). Data are presented as mean ±SEM. ^aaa^ *P* value <.001, the comparison between control and vehicle plus AF64A. ^###^ *P* value < .001, the comparison between vehicle plus ACSF and vehicle plus AF64A. **P value < .01, compared to vehicle + AF64A.

**Figure 2:**
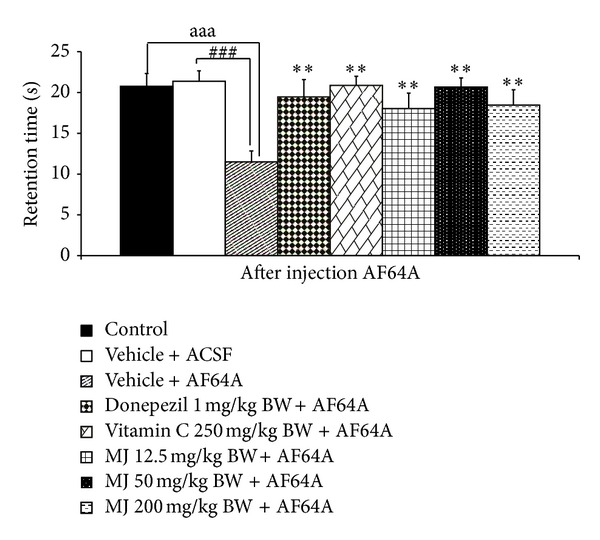
Effect of alcoholic extract of *M. indica* fruit on retention time in Morris water maze test (*n* = 8). Data are presented as mean ± SEM. ^aaa^ *P* value < .001, the comparison between control and vehicle plus AF64A. ^###^ *P* value < .001, the comparison between vehicle plus ACSF and vehicle plus AF64A. **P value < .01, compared to vehicle + AF64A.

The effect of *M. indica *fruit extract on spatial memory was shown in Figures 1 and 2. It was revealed that, when compared to control or sham operation + vehicle treated group, rats in vehicle + ACSF which received vehicle or distilled water and were subjected to the bilateral administration of ACSF showed no significant changes on both escape and latency times. Rats which received vehicle plus the bilateral administration of AF64A significantly increased escape latency but decreased retention time (*P* value < .001 all; compared to vehicle + ACSF). Both donepezil and vitamin C treated groups significantly attenuated the elevation of escape latency (*P* value < .01 all, compared to vehicle + AF64A) and the decreased retention time induced by AF64A (*P* value <.01 all; compared to vehicle + AF64A). Interestingly, our data also showed that the extract at all doses used in this study also could attenuate the enhanced escape latency and the decreased retention time induced by AF64A (*P* value < .01 all, compared to vehicle + AF64A).

### 3.2. Effect of *M. indica *Fruit Extract on Cholinergic System

**Figure 3:**
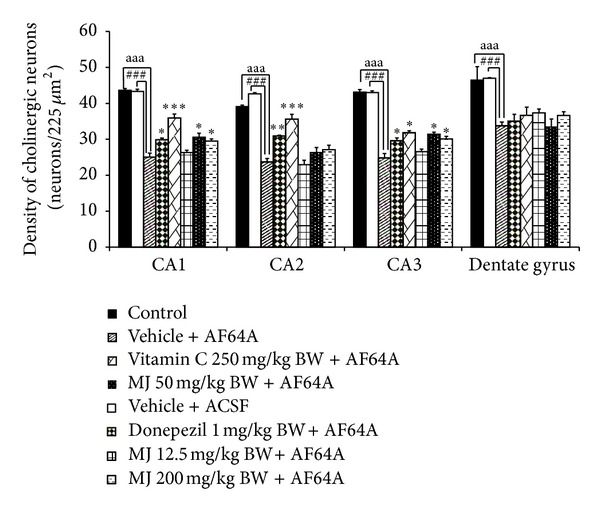
Effect of alcoholic extract of *M. indica* fruit on the density of cholinergic neurons in hippocampus (*n* = 8). Data are presented as mean ± SEM. *, **, ***P value < .05, .01, and .001, respectively, compared to vehicle + AF64A.

On the basis of the crucial roles of cholinergic system on learning and memory and the pathophysiology of MCI, we also investigated the effect of the extract on cholinergic system using the density of cholinergic neurons as indicator. The results were shown in Figure 3. When compared to control or rats subjected to vehicle treatment and sham operation, rats subjected to vehicle treatment and the bilateral administration of ACSF showed no significant changes in cholinergic neuron density in CA1, CA2, CA3, and dentate gyrus.

Rats which received vehicle plus AF64A showed the decreased cholinergic neurons density in CA1, CA2, CA3, and dentate gyrus (*P* value < .001 all, compared to vehicle plus ACSF). It was found that rats which received donepezil or vitamin C revealed the enhanced cholinergic neuron density in CA1 (*P* value < .05 and .01, respectively, compared to vehicle + AF64A), CA2 (*P* value < .01 and .001, respectively, compared to vehicle + AF64A), and CA3 (*P* value < .05 all, compared to vehicle + AF64A). In addition, rats subjected to the extract at doses of 50 and 200 mg·kg^−1^ BW also attenuated the decreased cholinergic neuron density induced by AF64A in CA1 and CA3 (*P* value < .05 all, compared to vehicle + AF64A). No other changes were observed.

### 3.3. Effect of *M. indica *Fruit Extract on Oxidative Stress Markers

**Figure 4:**
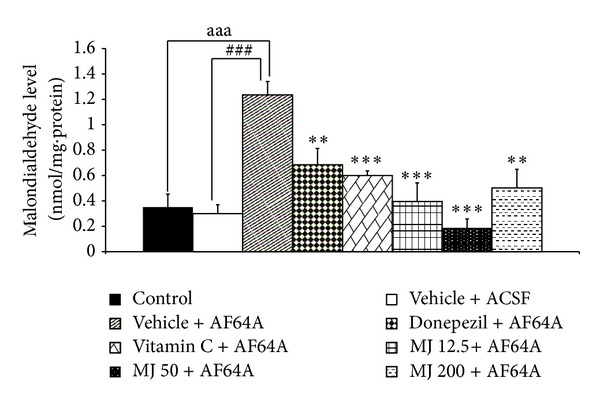
Effect of alcoholic extract of *M. indica* fruit on the level of malondialdehyde (MDA) in hippocampus (*n* = 8). Data are presented as mean ± SEM. ^aaa^ *P* value < .001, the comparison between control and vehicle plus AF64A. ^###^ *P* value < .001, the comparison between vehicle plus ACSF and vehicle plus AF64A. *, **, and ***P value < .01 and .001, respectively, compared to vehicle + AF64A.

**Figure 5:**
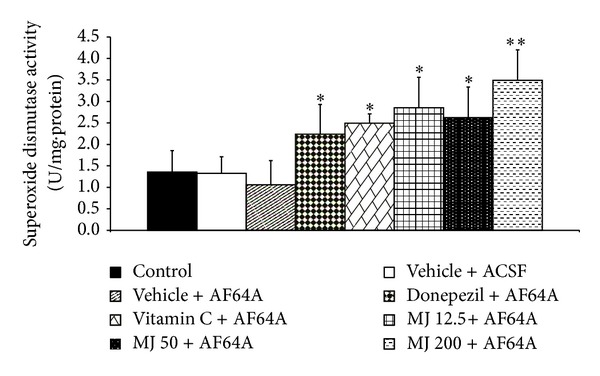
Effect of alcoholic extract of *M. indica* fruit on the activity of superoxide dismutase (SOD) in hippocampus (*n* = 8). Data are presented as mean ± SEM. *, ***P value < .05 and .01, respectively, compared to vehicle + AF64A.

**Figure 6:**
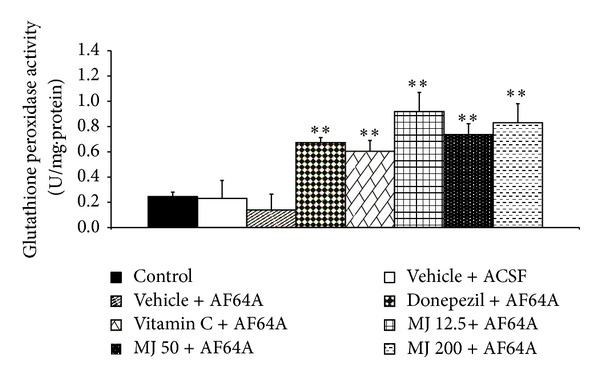
Effect of alcoholic extract of *M. indica* fruit on the activity of glutathione peroxidase (GSH-Px) in hippocampus (*n* = 8). Data are presented as mean ± SEM. **P value <.01 all, compared to vehicle + AF64A.

**Figure 7:**
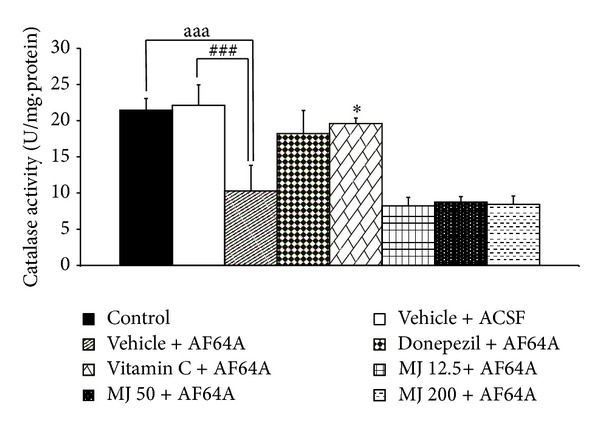
Effect of alcoholic extract of *M. indica* fruit on the activity of catalase (CAT) in hippocampus (*n* = 8). Data are presented as mean ± SEM. ^aaa^ *P* value <.001, the comparison between control and vehicle plus AF64A.^###^ *P* value <.001, the comparison between vehicle plus ACSF and vehicle plus AF64A. *P value <.05, compared to vehicle + AF64A.

The effects of *M. indica* fruit extract on oxidative stress markers including MDA level and the activities of SOD, CAT, and GSH-Px enzymes in hippocampus were also investigated and results were shown in Figures 3, 4, 5, 6, and 7. Figure 3 showed that, when compared to control or sham operation plus vehicle treatment, rats which were treated with vehicle plus ACSF did not show the significant changes of all parameters just mentioned. Rats which received vehicle plus AF64A produced a significant elevation of MDA level (*P* value < .001, compared to both vehicle plus ACSF and control groups) together with the decreased CAT activity in hippocampus (*P* value < .001, compared to both vehicle plus ACSF and control groups). No changes in SOD and GSH-Px activities in the mentioned area were observed in rats which received vehicle plus AF64A. It had been demonstrated that rats which obtained donepezil plus AF64A and vitamin C plus AF64A could decrease MDA level (*P* value < .01 and .001, resp.). Both rats which received donepezil plus AF64A and rats which received vitamin C plus AF64A showed the enhanced SOD (*P* value < .05 all, compared to vehicle plus AF64A) and GSH-Px activities (*P* value < .01 all, compared to vehicle plus AF64A) in hippocampus. In addition, rats subjected to Vitamin C treatment plus AF64A also enhanced CAT activity in hippocampus (*P* value < .05, compared to vehicle plus AF64A). Rats which received all doses of extract attenuated the enhanced MDA level (*P* value < .001, .001, and .01, resp., compared to vehicle plus AF64A) together with the decreased SOD (*P* value < .05, .05, and .01, resp., compared to vehicle plus AF64A) and GSH-Px activities in the area just mentioned (*P* value < .01, compared to vehicle plus AF64A).

## 4. Discussion

The current study has demonstrated that the alcoholic extract of *M. indica* significantly improves memory impairment together with the decreased oxidative stress and increased cholinergic neurons density in hippocampus.

It has been well known that spatial memory is the hippocampal dependent memory. Its capacity is associated with the neurodegeneration in hippocampus [13–15]. In this study, we have demonstrated the decreased cholinergic neuron density in CA1,CA2, CA3, and dentate gyrus of hippocampus together with the poor spatial memory in the hypocholinergic rats induced by AF64A. Therefore, our data are in agreement with the previous findings which showed that cholinergic deficit could induce memory impairment, while the increased cholinergic function especially in hippocampus could enhance spatial memory [13–16]. The current data also demonstrated that *Mangifera indica* extract at all doses used this study could improve memory impairment induced by AF64A but only the medium and high doses of extract could attenuate the decreased cholinergic neuron density in hippocampus. These data pointed out that the improved memory impairment might occur partly via the increased cholinergic neurons density in hippocampus, the area which played an important role on learning and memory. However, other factors such as the enhanced cerebral blood flow [17], the decreased brain oxidative stress status [18], and the alterations of other neurotransmitters including serotonin, catecholamine [19], GABA [20], and glutamate [21] might also contribute to the pivotal role in improved learning and memory. Therefore, the cognitive enhancing effect of low dose of extract might be associated with the changes of the mentioned parameters.

Our data also showed that no tight association between the increased cholinergic neurons density in hippocampus and the decreased MDA level in hippocampus was observed. Based on this finding, we did suggest that the decreased MDA level might not play a role only in the improved cholinergic neuron density in hippocampus but might also play a role in the increased neuron density of other neurochemical systems such as glutamate. However, this required further exploration.

Taking all data together, the cognitive enhancing effect of *M. indica* extract especially at medium and high doses might occur partly via the increased SOD and GSH-Px activities which in turn gave rise to the decreased MDA level in hippocampus. The decreased oxidative stress in turn induced the increased cholinergic neurons density and resulted in the improved spatial memory. However, the other factors contributing to the role in learning memory including the enhanced cerebral blood flow and the alteration of other neurochemical systems still cannot be eliminated.

Different susceptibility among various subregions in hippocampus was also observed. The possible explanation might be associated with the different distribution of neuronal system such as cholinergic neurons and granule cells which showed different vulnerability to the injury. The different distribution of growth factor, metabolic activity [16], and amount of blood supply [17] among various regions might also play roles.

In this study, we also failed to demonstrate the dose response manner of the extract. This might be attributed to the lack of simple relationship between the concentrations of extract and the observed parameters. In addition, the masking effect of inactive ingredients in the extract might also be possible because the extract which used in this study was the crude extract.

It has been demonstrated that gallic acid, a polyphenolic compound, is a ferric (Fe^3+^) chelator which in turn can attenuate the oxidative damage [22]. In addition, recent findings have shown that long term administration of beta-carotene also improves cognitive decline in men [23]. Therefore, it was also possible that the neuroprotective and cognitive enhancing effects of the extract might be associated with the phenolic and beta-carotene contents or the interaction of various active compounds in the *M. indica* fruit extract. The precise understanding still required further investigation.

## 5. Conclusion

This study is the first study to demonstrate that *M. indica* fruit extract is the potential neuroprotectant and cognitive enhancer for MCI in animal model. The possible actions may occur partly via the decreased oxidative stress and the increased cholinergic function. However, further researches are still required to elucidate the active ingredients and detail underlying mechanism.
